# Mixed-methods evaluation of targeted leadership development training to support the career progression of the black and minority ethnic NHS workforce

**DOI:** 10.1136/leader-2024-001175

**Published:** 2025-07-01

**Authors:** Holly Blake, Keir Scarlett, Sala Kamkosi Khulumula, Niki Chouliara

**Affiliations:** 1 School of Health Sciences, University of Nottingham, Nottingham, UK; 2 NIHR Nottingham Biomedical Research Centre, Nottingham, UK; 3 School of Medicine, University of Nottingham, Nottingham, UK; 4 Learning & Leadership Development, East London NHS Foundation Trust, London, UK; 5 Institute of Care Excellence, Nottingham University Hospitals NHS Trust, Nottingham, UK; 6 School of Sport, Exercise and Health Sciences, Loughborough University, Loughborough, UK

**Keywords:** career, career development, continuous improvement, Senior medical leader

## Abstract

**Introduction:**

Black and minority ethnic (BME) workers within the National Health Service (NHS) experience barriers to attainment of senior leadership roles. The NHS Leadership Academy delivered two Leadership Programmes addressing barriers to progression among the BME workforce, the Stepping Up (middle managers) and Ready Now (senior leaders) programmes.

**Methods:**

Mixed-methods evaluation involving an online survey (n=39; 20m/19f, identifying with 10 ethnic groups) and qualitative interviews (n=8; 5m/3f) with programme participants investigating barriers and the extent to which targeted leadership programmes impacted their career progression. Analysis included descriptive statistics and thematic analysis of qualitative data.

**Results:**

Participants reported institutional barriers to career progression and experiences of structural racism. Evaluation of targeted leadership programmes was consistently positive, providing a ‘safe space’ for shared experiences, while building self-confidence and motivation to apply for career development opportunities or positions. Most participants secured meaningful career development following programme completion.

**Conclusions:**

Targeted career development programmes are highly valued by BME healthcare workers and are perceived to contribute to addressing workforce inequalities in career progression relating to ethnic disparity in the attainment of senior NHS leadership roles. However, the broader impact of such programmes remains limited without addressing wider institutional barriers to progression and tackling racialised workplace inequities.

## Introduction

 Over one in five staff working in the National Health Service (NHS) in England are from black and minority ethnic (BME) backgrounds (22.4%^1^), but only 10.3% of those in senior management roles are BME workers, who are over-represented in less senior roles (34.5% at band 5).^2^ Many BME staff believe that their employer does not provide equal opportunities to career progression and promotion, compared with their white colleagues, specifically in relation to senior leadership roles.^1^ Indeed, white applicants are 1.61 times more likely to be shortlisted for job vacancies compared with BME applicants.^1^ The NHS Race and Health Observatory outlines the need to accelerate action to diversify its senior leadership.^3^ The NHS Leadership Academy has delivered two Leadership Programmes specifically targeted at BME staff, ‘Stepping Up’ aimed at middle managers, Bands 5–7 and ‘Ready Now’ suitable for senior leadership roles, Bands 8+. These programmes aimed to create greater levels of sustainable inclusion within the NHS by addressing social, organisational and psychological barriers restricting BME colleagues from progression. Stepping Up^4^ was delivered over 5 days in total, attended face to face, over 2–3 months. There were approximately six cohorts per year, delivered between 2014 and 2019. Ready Now^5^ consisted of 12 residential dates spread across 5 modules. There were approximately five cohorts per year, delivered between 2014 and 2019. This study aimed to investigate barriers to career progression, experiences of alumni relating to the leadership programme they attended, and any perceived impacts of the programme on their career progression.

## Methods

This mixed-methods study involved an online survey with closed and open-ended questions (phase 1: QUANT+QUAL), and individual qualitative interviews (phase 2: QUAL). Data were integrated to address the study aim. Study reporting was guided by the ‘Good Reporting of A Mixed Methods Study’ framework developed by O’Cathain *et al*.^6^ This aims to enhance the quality of reporting through a set of six reporting guidelines.^6^ Mixed-methods research was selected as it provides the opportunity to represent a greater diversity of views. Using an existing typology of research purposes,^7^ the study purpose was to ‘add to the knowledge base’ and ‘understand a complex phenomenon’, with study findings having potential to ‘have a personal, social, institutional and/or organisational impact’. Individuals who had taken part in all cohorts of the Stepping Up or Ready Now leadership development programmes between 2014 and 2019 (total n=990) were invited to take part in the study. Stepping Up consisted of two modules, lasting 3 months each. Ready Now was a year-long programme split across five modules. There were 40 individuals per cohort in each programme. The programme content and delivery format remained the same year-to-year. Here, we adopt the terminology used in the programmes, (‘black and minority ethnic: BME’), but this also reflects ‘BAME’, ‘ethnically minoritised communities’ or ‘global majority’. This was a convenience sample. Invitations were sent by email via the NHS Leadership Academy in September 2023 and contained the study information sheet and a link to an anonymous online survey hosted on Microsoft Forms (online supplemental file 1). A single reminder was sent 7 weeks later. Survey completers could consent to take part in an online semistructured qualitative interview (online supplemental file 2) with the study researcher, on Microsoft Teams, to share their views in more depth. The survey included demographic items, views towards BME-targeted programmes, and items relating to the programme attended, perceived benefits of attending, and any career progression since programme completion. Response options included categorical (yes/no) items, free text and 5-point Likert-type scales: ‘extremely useful’, ‘very useful’, ‘somewhat useful’, ‘slightly not useful’ and ‘not at all’. Quantitative survey data were analysed using IBM SPSS Statistics (V.27). Interviews were audio recorded, transcribed and anonymised. Inductive thematic analysis^8^ was applied to the two qualitative data sets collected from free-text responses in the online survey (phase 1) and interviews (phase 2) to enable the identification and analysis of notable themes or patterns in the data. There were six steps of thematic analysis conducted as part of this process: familiarisation with the data, the creation of a coding system, coding data and identifying key themes, reviewing and defining notable themes, and then analysis of those themes. Qualitative data were analysed using MAXQDA Analytics Pro V.24.4.1, which allows for a ‘high degree of reflexivity’^9^ in the organisation and coding of data. MAXQDA allowed data from multiple documents to be compiled and coded as individual themes emerged, using functionality such as memos and hyperlinks between multiple documents. Through this process, themes were gradually condensed and organised into a more refined group of thematic categories which demonstrated an emerging narrative to the participant experience.

## Results

**Figure 1:**
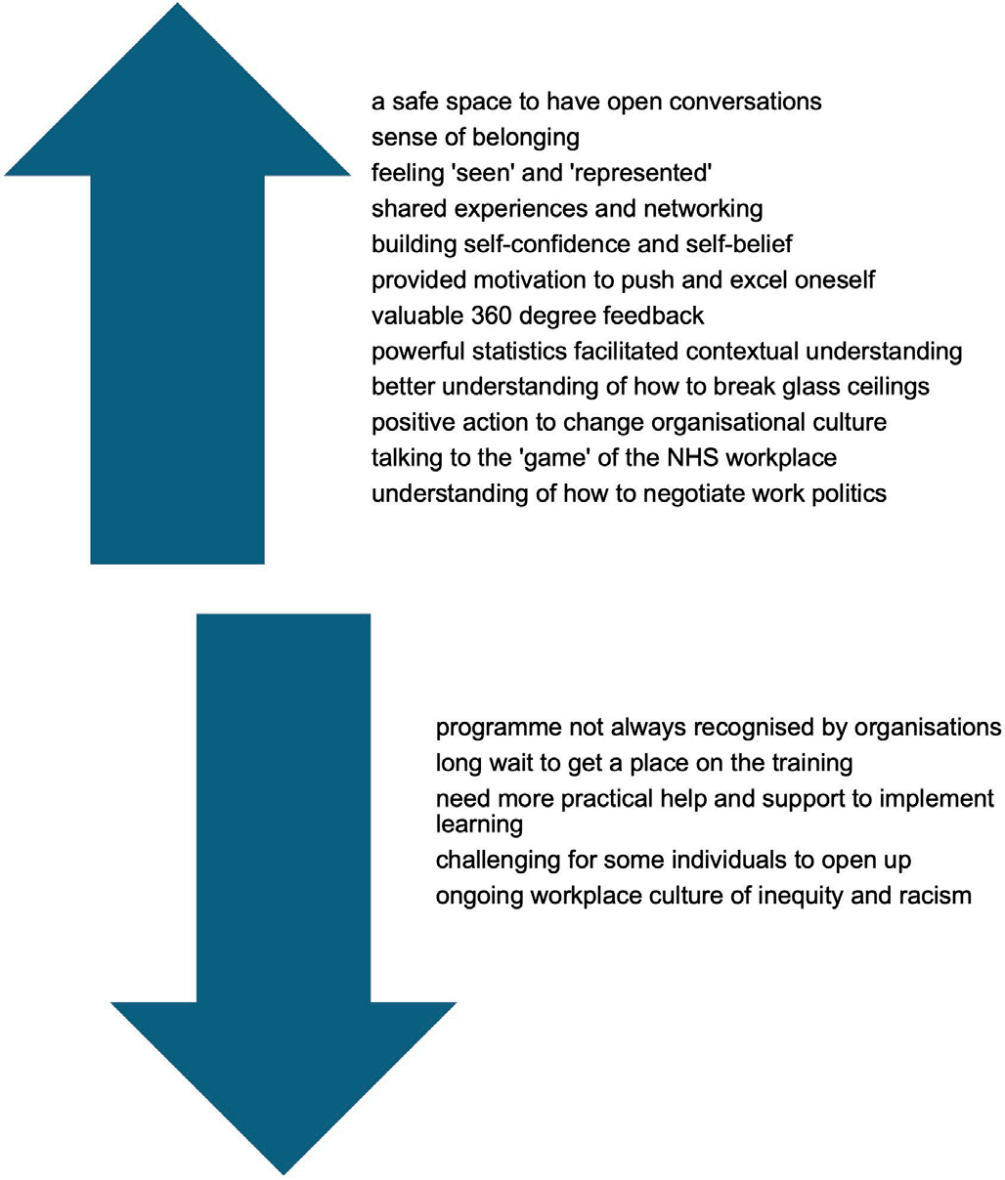
Views towards the benefits and challenges of the programme. NHS, National Health Service.

39 individuals (20 male, 19 female) who had attended the Stepping Up (n=20) or Ready Now (n=19) programmes between 2014 and 2019 completed the survey. All were heterosexual, seven had a disability. They were aged 35–44 (n=14, 36%), 45–54 (n=19, 49%) and 55–64 years (n=6, 15%) and identified with 10 different ethnicities: Asian/Asian British–Indian (n=13, 33%), Black/Black British–African (n=7, 18%), Black/Black British–Caribbean (n=7, 18%), Asian/Asian British–Pakistani (n=5, 13%), Mixed–White and Black African (n=1, 2.5%), Asian/Asian British–Chinese (n=1, 2.5%), Mixed–White and Black Caribbean (n=1, 2.5%), White other–Slovakian (n=1, 2.5%), Japanese/Korean/Chinese (n=1, 2.5%), Asian British–Other (n=1, 2.5%). All except one found the programme useful (‘transformational’, ‘empowering’, ‘a self-discovery journey’, ‘life-changing experience’, ‘the best training and development experience of my whole professional career’, ‘can’t rate highly enough’) and reporting having put their learning into practice (n=38, 97%). Most participants believed a targeted programme was better than generic programmes (n=34, 81%), would recommend targeted leadership programmes to colleagues (n=36, 92%) and had shared their learning with BME colleagues (n=36, 92%). Reported benefits of the programme included: it being a ‘safe space’ (n=14, 36%), with tailored content to BME experiences (n=9, 23%), networking opportunities (n=8, 20.5%), exposure to other opportunities (n=5, 13%) and the wide recognition of these NHS leadership programmes among employers (n=2, 5%). Views towards the programme are shown in figure 1.

Mixed-methods analysis identified five themes relating to the influencers of career progression and outcomes of the programme(s) (table 1).

**Table 1:** Themes and illustrative quotes

Theme	Illustrative quotes
Theme 1: Institutional barriers to career progression	“Whilst I didn’t greatly benefit within the Trust, I think my boss used it as a flag to bolster their credentials” (Asian British—other, female, 45–54: RN) “It was the White staff that went on programmes… BME staff really struggle to get even time off work to be able to do those programmes” (Mixed—White and Black African, male, 35–44, SU) “I have the ability not to be funnelled into EDI [roles]… I am capable as a systems leader or an operational leader” (Asian British – other, female, 45–54, RN) “I don’t think organisations appreciate the value of it. I have come back, and I’m still pushed into leadership courses of a lesser standard” (Asian British – other, female, 45–54, RN) “You’re just not seen at the same trajectory, and also there’s thing of – oh you’ve for to an 8a, that’s good enough for you” (Mixed—White and Black African, male, 35–44, SU) “Go on this course, it’ll keep you quiet for a while” (Asian British—other, female, 45–54, RN) “When people of colour apply, they not only meet the criteria… they might have more than enough relevant experience that’s completely discounted” (Mixed—White and Black African, male, 35–44, SU) “They(BME-individuals)are not put forward, and even when they do get through, they’re not selected because somebody is a better because of where they’re from” (Mixed—White and Black African, male, 35–44, SU), “Internally, every no is dismissive and indicates my face does not fit in unless something is needed for Black History Month” (Black British African/Caribbean, male, 45–54, RN) “If a colleague experiences racism, it may be that a patient experiences racism as well” (Asian/Asian British—Indian, male, 45–54, RN)
Theme 2: Role of line managers in career progression	“When you go on the programme you have a sponsor within the trust, I think more should be asked of those sponsors… exposing you to opportunities… giving you access to higher level meetings, allowing you to sit and shadow them” (Asian British—other, female, 45–54, RN) “They need to target band 6 and 7, because there are people in those bands who’ve nursed for 20–30 years and are still stuck in those bands” (Mixed – White and Black African, male, 35–44, RN) “(I)had to convince my manager to release me to go… and if I didn’t look into it myself, I wouldn’t have been put forward” (Mixed—White and Black African, male, 35–44, SU) “Every now and then I’m thrown a bone to keep me quiet” (Asian British—other, female, 45–54, RN) “I left the programme feeling hopeful and I realised that I was no longer under the mercy of my manager if I felt I was ready to step up in my career” (Black/Black British—African, female, 35–44, SU)
Theme 3: Creating psychological safety	“To understand the impact of such trauma in the landscape and how to push beyond” (Asian British—other, female, 45–54, RN) “Being in a room full of like-minded people where you have experienced gaslighting… it’s not just you, you don’t need to feel paranoid” (Asian British—other, female, 45–54, RN)
Theme 4: Instilling confidence in oneself	“It gave me the confidence to put myself in the shop window, because if I hadn’t, I wouldn’t have had the opportunities I have had” (Black British African/Caribbean, male, 45–54, RN) “[The programme showed me] here’s how I go forward, and I could do these things and [there are opportunities I can] put myself forward for” (Asian British—other, female, 45–54, RN) “I was disillusioned with the NHS, and even though I had my experience, and I was a good nurse, all the bad experiences kind of lost a bit of [confidence]. I think this course really motivated me, and started the motivation again” (Black British African/Caribbean, female, 45–54, RN) “Externally, every no leads me closer to a yes [at interview] with support and coaching” (Black British African/Caribbean, male, 45–54, RN)
Theme 5: Tangible career progression	“Because of that… I have moved significantly from where I was when I went on the programme… I am now an Interim Chief Allied Health Professional” (Black/Black British—African, male, 45–54, RN) “Out of nine of us who started the programme, about four of us have already got into senior jobs” (Asian British—other, female, 45–54, RN) “Within 6 months, I secured another job which was a career progression for me” (Black/Black British—African, female, 45–54, SU).

### Theme 1: institutional barriers to career progression

There were accounts of institutional racism, challenges associated with getting time off work to attend training or access senior level programmes compared with white colleagues, perceptions of being passed over for promotion or ‘sidelined’ into lower level or equality, diversity and inclusion roles.

### Theme 2: role of line managers in career progression

There were mixed experiences of line manager support, some participants felt dismissed or recipients of ‘lip service’ where progression opportunities were concerned. Participants advocated targeted approaches led by line managers, such as shadowing, mentoring and talent spotting at Bands 6 and 7.

### Theme 3: creating psychological safety

Some participants had experienced ‘gaslighting’ leading to self-doubt in their capability. The programmes created a ‘safe space’ for sharing experiences and strategies to support career development and progression.

### Theme 4: instilling confidence in oneself

Most participants reported increased self-confidence after attending the programme, and subsequently, were more motivated to take actions relating to personal development and career progression.

### Theme 5: tangible career progression.

Of survey respondents, 34 (87%) believed that attending the programme positively impacted their career progression. Participants reported that following the programme they ‘progressed in their career’ (n=24/39, 62%), changed career/job role with a ‘sideways step’ (n=5/39, 13%), remained in their role (n=6/39, 15%) or had another outcome (eg, retirement) (n=4/39, 10%). This was reflected in the interview data, where many participants reported having transitioned into senior job roles.

## Discussion

These targeted leadership programmes delivered by the NHS Leadership Academy^4 5^ were perceived to be highly successful in providing personal development and progressing the careers of NHS staff from BME backgrounds. Many participants had changed job roles, moved to other institutions or into roles that offered more flexibility and autonomy and/or progressed into more senior positions. All participants reported positive impacts on their career, including those who had remained in the same job role. There were many benefits of attendance, but the creation of a safe space in which BME individuals could share experiences, speak freely and feel recognised was the most prominent. For some, safe spaces were sustained postprogramme via WhatsApp groups, creating ongoing opportunities for community building and networking.

Mixed-methods analysis allowed greater insights and perspectives than one method alone, which is valuable given the lack of prior research on this topic. There are several study limitations. Responses were gathered from a small pool of programme attendees due to the time and resource impacts of data integration in a sequential mixed methods study. The views of those who could not be successfully contacted (eg, non-functioning email address), or chose not to respond, are not known. Asian/Asian British Indian participants were over-represented in the sample. Due to the small sample size, we were unable to explore participants’ experience in the context of sex, disability or sexual orientation or the influence of intersectionality of participant identities on their perceptions and views.

Nonetheless, attendance on the programmes increased participants’ confidence and motivation for personal development, which are ‘important determinants of success’ in career progression.^10 11^ Those with high levels of confidence are often more motivated to overcome obstacles and take beneficial risks^12^ which may increase the likelihood of career progression.

However, there is widespread concern for the institutional barriers that remain and hinder progression for BME individuals. Some participants reported a sense of ‘career plateauing’, a lack of managerial support for development opportunities, fear of the impact of an institutionally racist healthcare system on patients and intentions to leave the NHS. The ‘glass ceiling’ of institutional racism on career progression is well documented^1 13^ and manifests in the devaluation and disregard for the experience, qualifications and potential of individuals from a BME background. These findings align with prior research suggesting that ‘organisations create a segregated and racialised career experience’ for those from minoritised ethnic groups, which holds back careers.^14^

Due to financial limitations, these programmes have ceased, and many Trusts offer generic in-house training instead, although quality and access can be variable. This may hinder the development of future BME leaders. Our mixed-methods evaluation of Stepping Up and Ready Now was consistently positive. The programmes were highly valued by attendees with unique benefits extending beyond generic leadership training. The ethos of targeted leadership programmes clearly aligns with the recommendation for embedding race across development opportunities and diversifying senior leadership.^3 15^ However, the broader impact of such leadership programmes remains limited without addressing wider institutional barriers to progression and tackling racialised workplace inequities.

## Supplementary material

10.1136/leader-2024-001175 online supplemental file 1
