# Supplementary material for: Mixed-methods evaluation of targeted leadership development training to support the career progression of the black and minority ethnic NHS workforce
Source: BMJ Lead. 2025 Jul 1;9(4):e001175. doi: 10.1136/leader-2024-001175 (PMC12772536; doi:10.1136/leader-2024-001175)
Supplement: online supplemental file 1 [file leader-9-4-s001.pdf]

Supplementary file 1: Online survey

# Evaluating the effectiveness of targeted development programmes on BME career development

This online survey has been designed for the purposes of research into: Evaluating the effectiveness of targeted Leadership Development Programmes on career development from the perceptions of Black and Minority Ethnic workforce within the NHS.

Responses to this survey are anonymous, and individuals are encouraged to read the Participant Information Sheet and Consent Form attached to the email containing this survey link, prior to participating.

* Required

1. Please identify the title of the programme you completed with the NHS Leadership Academy *

- ☐ Stepping Up Leadership Programme
- ☐ Ready Now Leadership Programme

2. In which year did you complete your Leadership Programme? - Only year is taken into account *

3. In your view, how useful to your current role was the content from the Programme? *

- ◯ Extremely useful
- ◯ Very useful
- ◯ Somewhat useful
- ◯ Slightly not useful
- ◯ Not at all

4. Has your completion of the Programme benefitted you in your personal or professional development? *

- ◯ Yes
- ◯ No

5. How do you feel your completion of the Stepping Up or Ready Now Programme, has benefitted you as an individual in your development? *

6. Please tell us more about your previous answer: *

7. How tailored to the experiences of black and minority ethnic individuals, do you consider the content of the programme to be? *

- ○ Greatly
- ○ Somewhat
- ○ Not very
- ○ Not at all

8. Have you put your learning and development from the Programme into practice? *

- ○ Yes
- ○ No

9. Please tell us more about your answer: *

10. Since completing the Leadership Programme/s, have you: *

- ○ Progressed in your career
- ○ Taken a sideways move
- ○ Remained in your current role
- ○ Other

11. If you have remained in your current role, what do you feel is the reason for this? *

- ◯ Lack of opportunities
- ◯ Unsuccessful at interview
- ◯ Lack of practical experience
- ◯ Content in current role
- ◯ Personal circumstance
- ◯ No desire to progress
- ◯ Other

12. Do you feel your completion of the Leadership Programme/s positively contributed to you being successful in promotion or sideways career move? *

- ◯ Yes
- ◯ No
- ◯ Unsure

13. Please tell us more about your answer: *

14. Do you feel your experience on the BME-Targetted Leadership Programmes was more effective or useful, than it would have been on a generic Leadership Programme *

- ◯ Yes
- ◯ No
- ◯ Unsure

15. Please tell us more about your previous answer *

16. In your own words, how would you describe your experience during, and following the Stepping Up or Ready Now Leadership Programmes? *

17. What would you consider the main benefit to your completion of the Programme? *

- ◯ Networking with other BME colleagues
- ◯ Provided with a safe space to grow/share
- ◯ Recognised NHS qualification
- ◯ Content relevant to BME experiences
- ◯ Exposure to further opportunities for career development
- ◯ Other

18. Have you shared your learning from the Programme with other BME colleagues? *

- ◯ Yes
- ◯ No

19. What is the reason for this? *

20. How have you done this? Do you feel it has had a positive impact on your BME colleagues? *

21. Are you a member of your Organisation's BME Staff Network? *

- ◯ Yes
- ◯ No

22. If you are not currently a member of your Organisation's BME Network, please tell us why: *

23. Would you recommend development programmes, specifically-targeted at BME communities to a colleague? *

- Yes
- No
- Unsure

24. Please provide a rationale for your answer: *

25. As part of this research, we are inviting individuals to take part in an additional interview on Microsoft Teams. The purpose of these interviews are to further explore your survey responses and allow you to greater contextualise or expand on your answers.

The interview will last approximately 45 minutes.

If you would like to volunteer to participate in a follow-up interview, please provide a contact email address below.

Please note that this will be stored securely and used solely for the purpose of contacting you to arrange a suitable interview date/time. Personal information will not be used within research findings or used to identify individual responses.

If you are not interested in participating in an interview, please feel free to skip this question.

26. Please select your age group: *

- 18-24
- 25-34
- 35-44
- 45-54
- 55-64
- 65+

27. Please select your ethnicity ( selecting one of the below options will prompt additional options) *

- ○ White - Welsh / English / Scottish / Northern Irish / British
- ○ White - Irish
- ○ White - Gypsy or Irish Traveller
- ○ Mixed - White and Black Caribbean
- ○ Mixed - White and Black African
- ○ Mixed - White and Asian
- ○ Asian/Asian British - Indian
- ○ Asian/Asian British - Pakistani
- ○ Asian/Asian British - Bangladeshi
- ○ Asian/Asian British - Chinese
- ○ Black/Black British - Caribbean
- ○ Black/Black British - African
- ○ Arab
- ○ Other

28. Please select which gender you identify as: *

- ○ Male
- ○ Female
- ○ Transgender Male
- ○ Transgender Female
- ○ Prefer not to say
- ○ Other

29. Please select your sexual orientation *

- ○ Heterosexual
- ○ Homosexual
- ○ Bisexual
- ○ Other

30. Do you have a disability? *

- ○ Yes
- ○ No
- ○ Prefer not to say

This content is neither created nor endorsed by Microsoft. The data you submit will be sent to the form owner.

Microsoft Forms

**Interview Questions**

1. Please explain your experience on the Development Programme you attended

2. How do you think the programme contributed to your personal or career development? What do you think the reasons are for this?

3. What has been your experience following your completion of the Programme? Have you been given opportunities to put learning into practice and develop further?

4. Do you think the development programme could be made even more impactful to your development? If so, how?

5. Have you remained in contact with any networking connections made on the Programme?

6. Would you consider attending BME-specific Development Programmes in future? Or recommend them to a colleague?

7. Do you think further BME-focused Diversity Programmes should be welcomed into the NHS?
